# Mesenchymal stromal cells of osteosarcoma patients do not show evidence of neoplastic changes during long-term culture

**DOI:** 10.1186/s13569-015-0031-1

**Published:** 2015-06-23

**Authors:** Emilie P Buddingh, S Eriaty N Ruslan, Christianne M A Reijnders, Karoly Szuhai, Marieke L Kuijjer, Helene Roelofs, Pancras C W Hogendoorn, R Maarten Egeler, Anne-Marie Cleton-Jansen, Arjan C Lankester

**Affiliations:** Department of Pediatrics, J6-S, Leiden University Medical Center, PO Box 9600, 2300 RC Leiden, The Netherlands; Department of Pathology, Leiden University Medical Center, Leiden, The Netherlands; Department of Molecular Cell Biology, Leiden University Medical Center, Leiden, The Netherlands; Department of Immunohematology and Blood Transfusion, Leiden University Medical Center, Leiden, The Netherlands

## Abstract

**Background:**

In vitro expanded mesenchymal stromal cells (MSCs) are increasingly used as experimental cellular therapy. However, there have been concerns regarding the safety of their use, particularly with regard to possible oncogenic transformation. MSCs are the hypothesized precursor cells of high-grade osteosarcoma, a tumor with often complex karyotypes occurring mainly in adolescents and young adults.

**Methods:**

To determine if MSCs from osteosarcoma patients could be predisposed to malignant transformation we cultured MSCs of nine osteosarcoma patients and five healthy donors for an average of 649 days (range 601–679 days). Also, we compared MSCs derived from osteosarcoma patients at diagnosis and from healthy donors using genome wide gene expression profiling.

**Results:**

Upon increasing passage, increasing frequencies of binucleate cells were detected, but no increase in proliferation suggestive of malignant transformation occurred in MSCs from either patients or donors. Hematopoietic cell specific Lyn substrate 1 (*HLCS1*) was differentially expressed (fold change 0.25, *P* value 0.0005) between MSCs of osteosarcoma patients (n = 14) and healthy donors (n = 9).

**Conclusions:**

This study shows that although *HCLS1* expression was downregulated in MSCs of osteosarcoma patients and binucleate cells were present in both patient and donor derived MSCs, there was no evidence of neoplastic changes to occur during long-term culture.

**Electronic supplementary material:**

The online version of this article (doi:10.1186/s13569-015-0031-1) contains supplementary material, which is available to authorized users.

## Background

Mesenchymal stromal cells (MSCs) are increasingly used as experimental cellular therapy in a wide range of conditions, such as graft-versus-host disease in the context of allogeneic bone marrow transplantation, auto-immune diseases and for regenerative purposes in for example myocardial injury or cartilage defects [1–6]. However, since MSCs have to be expanded in vitro to achieve sufficient numbers, there have been concerns regarding the safety of their use, particularly with regard to possible oncogenic transformation [7]. Cultured murine MSCs readily transform and form sarcoma-like tumors in vivo [8–12]. Similarly, MSCs derived from rhesus macaques become polyploid and subsequently aneuploid during long-term culture [13]. In contrast, human MSCs appear resistant to spontaneous in vitro transformation [14]. Studies reporting that human MSCs undergo malignant transformation in vitro have been retracted because of cross-contamination issues [15–20]. Despite the apparent difference between human and murine MSCs in their propensity to spontaneously transform in vitro, concerns remain. MSCs are hypothesized to be the precursor cells of high-grade osteosarcoma (OS) and a patient transplanted with bone marrow (containing hematopoietic stem cells and MSCs) from a sibling was diagnosed with OS originating from donor stem cells 17 years later [21]. This case demonstrates that donor-derived (pre-) cancerous MSCs can survive in a host and cause disease many years later. Another cause for concern is the observation that cultured MSCs can acquire chromosomal aberrations, although these do not seem to confer a selective growth advantage in vitro [22, 23].

High-grade osteosarcoma is a malignant primary bone tumor which often occurs at a relatively young age [24]. OS tumor cells are characterized by aneuploid karyotypes and gross chromosomal instability [25]. Such highly complex chromosomal rearrangements can occur as a result of a single catastrophic event, termed chromothripsis [26, 27]. However, this probably has to occur in a susceptible background, either as a genetic predisposition or acquired as a de novo event. In a murine model failed cytokinesis can lead to tetraploidy and subsequent tumorigenesis only in a p53 deficient host [28]. We previously showed loss of CDKN2A/p16 protein expression in tetraploid tumorigenic murine MSCs [9]. We hypothesized that normal MSCs from OS patients could be predisposed to malignant transformation, and performed long term in vitro culture and genome wide expression profiling of early passage MSCs from OS patients and healthy donors. Here we show that OS patient-derived MSCs do not transform in vitro, confirming previous reports in healthy individuals that spontaneous transformation of human MSCs in vitro is an extremely unlikely event.

## Methods

### Patients

Characteristics of OS patients and healthy stem cell donors can be found in Table 1. Bone marrow cells of OS patients were harvested under general anesthesia prior to start of the chemotherapeutic treatment. The site of MSC harvest (iliac crest) was different from the location of the primary tumor (metaphyseal ends of the long bones) in all cases. Healthy donors were either identical sibling donors for patients with malignant or benign disease requiring hematopoietic stem cell transplantation; or haploidentical donors for either hematopoietical stem cell transplantation or the therapeutic infusion of MSCs for steroid-resistant graft-versus-host disease. Written informed consent was obtained from all patients and donors prior to bone marrow harvesting. The study was approved by the Institutional Review Board on Medical Ethics [LUMC Medical Ethics Committee (CME), P06.152].

**Table 1:** Characteristics of osteosarcoma patients (OS) and healthy donors (HD) and overview of experiments

Culture	OS (UI)/HD	Sex	Age (years)	Histology	Location of the primary tumor	Included in experiment		
						Microarray	qPCR	Long-term culture
001OS	OS (352)	M	12	Osteoblastic	Distal femur	Y	Y	Y
002OS	OS (340)	F	13	Osteoblastic	Distal femur	Y	Y	Y
003OS	OS (376)	F	13	Telangiectatic	Distal femur	Y	Y	Y
004OS	OS (377)	F	14	Sclerosing	Proximal tibia	Y	Y	Y
005OS	OS (348)	F	15	Telangiectatic	Distal tibia	N	N	Y
006OS	OS (349)	M	8	Osteoblastic	Distal femur	Y	Y	Y
007OS	OS (350)	F	15	Osteoblastic	Distal femur	N	N	Y
008OS	OS (378)	F	9	Osteoblastic	Proximal humerus	Y	Y	Y
009OS	OS (382)	M	15	Chondroblastic	Proximal humerus	Y	Y	Y
010OS	OS (388)	M	14	Osteoblastic	Proximal humerus	N	Y	N
011OS	OS (391)	F	14	Osteoblastic	Proximal tibia	N	Y	N
012OS	OS (394)	F	5	Osteoblastic	Distal femur	N	Y	N
013OS	OS (393)	F	14	Osteoblastic	Distal femur	N	Y	N
014OS	OS (395)	M	10	Sclerosing	Distal femur	N	Y	N
015OS	OS (396)	M	13	Osteoblastic	Distal femur	N	Y	N
016OS	OS (402)	F	15	Sclerosing	Proximal tibia	N	Y	N
HB	HD (HB)	M	15			Y	Y	Y
HD3	HD (HD3)	F	27			Y	Y	Y
HD5	HD (HD5)	M	50			Y	Y	Y
MH	HD (MH)	M	15			Y	Y	Y
TD1	HD (TD1)	F	11			Y	Y	Y
TD2	HD (TD2)	F	43			N	Y	N
TD3	HD (TD3)	F	43			N	Y	N
TD4	HD (TD4)	F	25			N	Y	N
TD5	HD (TD5)	M	5			N	Y	N

*UI* unique identifier.

### Mesenchymal stromal cell cultures

Bone marrow derived mononuclear cells were obtained from 5 to 15 mL of heparinized bone marrow aspirate by density gradient centrifugation on Ficoll. Cells were plated on non-coated 75 cm^2^ polystyrene flasks at a cell density of 160,000/cm^2^ in complete culture medium (LG-DMEM; Invitrogen, Paisley, UK) supplemented with penicillin/streptomycin (Invitrogen) and 10% fetal bovine serum (FBS; HyClone, Verviers, Belgium). We used a characterized and defined FBS batch preselected for its potential to support MSC expansion and continued to use this specific batch throughout the culture period. MSCs were plastic adherent and had spindle shaped morphology. Chondrogenic, adipogenic and osteoblastic differentiation were performed as described earlier [29]. Medium was refreshed twice a week and cells were replated when reaching 80–90% confluence at a density of 4,000/cm^2^. The first nine OS patient and first five healthy donor MSC samples that were obtained were cultured long-term, the subsequent samples were used for confirmatory mRNA expression analysis (Table 1). Morphology of the cells was recorded and population doublings per passage (PD) were calculated using the log ratio of the harvesting cell count (N) to the starting (baseline) count (X0), divided by the log of 2 (PD = [log (N/X0)]/log 2).

### Flow cytometry

Expression of the cell surface markers CD73, CD90 and CD105 and absence of hematopoietic markers on MSCs was determined using flow cytometry, according to the statement by the International Society for Cellular Therapy [30]. Cells were detached using Trypsin/EDTA and washed in PBS/0.05% bovine serum albumin. Antibodies used were (FITC) conjugated anti-CD86-fluorescein isothiocyanate (FITC) (cat. no. 555657), anti-HLA-DR-FITC (cat. no. 347400), anti-CD31-phycoerythrin (PE) (cat no 555446), anti-CD34-PE (cat no 348057), anti-CD73-PE (cat no 550257), anti-CD90-PE (cat no 555596), anti-CD3-peridinin chlorophyll protein(PerCP)-Cy5.5 (cat no 332771), anti-CD45-PerCPCy5.5 (cat no 332784), all from BD (San Diego, CA, USA) and anti-CD105-PE (cat no SN6), from Ancell (Bayport, MN, USA). Flow cytometry was performed on a FACScalibur, and analyzed using Cellquest software (both Becton–Dickinson). Mean fluorescence intensity (MFI) ratio was calculated by determining the MFI of the specific staining relative to the MFI of the appropriate isotype control staining.

### Analysis of binucleate cells

MSCs were grown on coverslips. Coverslips were washed with PBS and fixed in 4% PFA. Coverslips were incubated for 10 min in 1 μg/mL acridine orange (Sigma-Aldrich, Zwijndrecht, the Netherlands) to stain cytoplasm and 1 μg/mL wheat-germ agglutinin with an Alexa Fluor^®^ 594 conjugate (Invitrogen) in PBS to stain cell membranes. Following washes in PBS, coverslips were mounted using Vectashield with DAPI (Vector laboratories, Burlingame, CA, USA) to stain nuclei. Images were acquired using a COHU 4910 series monochrome CCD camera (COHU, San Diego, CA, USA) attached to a DM fluorescence microscope (Leica, Wetzlar, Germany) and analyzed using ImageJ software with the Cell Counter plug-in (National Institute of Mental Health, Bethesda, MD, USA).

### RNA isolation

RNA was isolated from frozen cell pellets of at least 1*10^e^6 undifferentiated MSCs at passage 2–5 using TRIzol reagent (Invitrogen). Cells were lysed in TRIzol, followed by phase separation in chloroform, precipitation using 2-propanolol and washing in 75% ethanol. RNA clean-up was performed using the QIAGEN Rneasy mini kit (Venlo, the Netherlands) with on-column DNAse treatment. RNA quality and concentration were measured using an Agilent 2100 Bioanalyzer (Santa Clara, CA, USA) and Nanodrop ND-1000 (Thermo Fisher Scientific, Waltham, MA, USA), respectively.

### cDNA synthesis, cRNA amplification, and Illumina Human v2.0 Expression BeadChip hybridization

Gene expression profiling was performed using Human-6 v2 Expression BeadChips (San Diego, CA, USA) containing >48,000 transcript probes. Synthesis of cDNA, cRNA amplification, and hybridization of cRNA onto the Illumina Human v2.0 Expression BeadChips were performed as described previously [31, 32].

### qPCR

cDNA synthesis for qPCR was performed as described earlier [32]. qPCR was performed using the iQ™ SYBR^®^ Green Supermix (Bio-Rad, Hercules, CA, USA) according to the manufacture’s instructions, reverse transcriptase negative (RT−) and H_2_O controls were taken along for each sample. For normalization, three genes with stable expression in the microarray experiments were chosen (*CPSF6*, *GPR108* and *CAPNS1*). Data were normalized by geometric mean expression levels of the three reference genes using geNorm (http://medgen.ugent.be/jvdesomp/genorm/). Primer sequences can be found in Additional file 1: Table S1. A standard curve was taken along for each primer set and used for quantification, PCR efficiencies ranged from 93 to 104%.

### Karyotyping

Six early passage samples (three derived from OS patients, three derived from healthy donors) were subjected to a multicolor FISH based karyotyping test (COBRA-FISH) as described earlier [33]. From each sample at least 20 metaphase cells were recorded and analyzed.

### Statistical analysis

Microarray data were normalized using the Cubic Spline normalization method with the Illumina BeadStudio Gene Expression Module. Microarray data are available at GEO using the accession no. GSE42572. Statistical analysis of microarray was performed using Significance Analysis for Microarrays, using a false discovery rate of 20% (SAM, http://www-stat.stanford.edu/~tibs/SAM/) [34]. Univariate statistical analyses were performed using GraphPad Prism software (version 5.01, La Jolla, CA, USA). Two-sided *P* values lower than 0.05 were determined to be significant.

## Results

### MSCs of OS patients and healthy donors do not differ in expression of cell surface markers or differentiation capacity

We tested the MSC cultures from OS patients and healthy donors for phenotypic markers and functionality. Samples from 16/16 patients and 9/9 controls were able to differentiate into chondrogenic (Additional file 2: Figure S1A), adipogenic (Additional file 2: Figures S1B and C) and osteoblastic lineages (Additional file 2: Figures S1D and E). Also, all MSC cultures expressed CD73, CD90 and CD105 and lacked expression of hematopoietic markers (Additional file 2: Figure S1F). Level of expression of CD73, CD90 and CD105 as determined by MFI-ratios (specific staining/isotype control) did not differ between MSC cultures derived from OS patients and healthy donors. Early passage samples from three OS patient derived MSCs and three healthy donor derived MSCs were karyotyped; no structural or numerical aberrations were observed in the analyzed samples (Additional file 3: Figure S2): MSC001: 46,XY (26 metaphases), MSC003: 46,XX (27 metaphases), MSC008:46,XX (22 metaphases), MSC-HB: 46,XY (21 metaphases), MSC-MH: 46,XY (20 metaphases), MSC-TD: 46,XX (21 metaphases). Late passage cells could not be karyotypically analyzed due to the low numbers of dividing cells.

### Long-term in vitro culture of MSCs results in increased binucleation but not in malignant transformation

**Figure 1:**
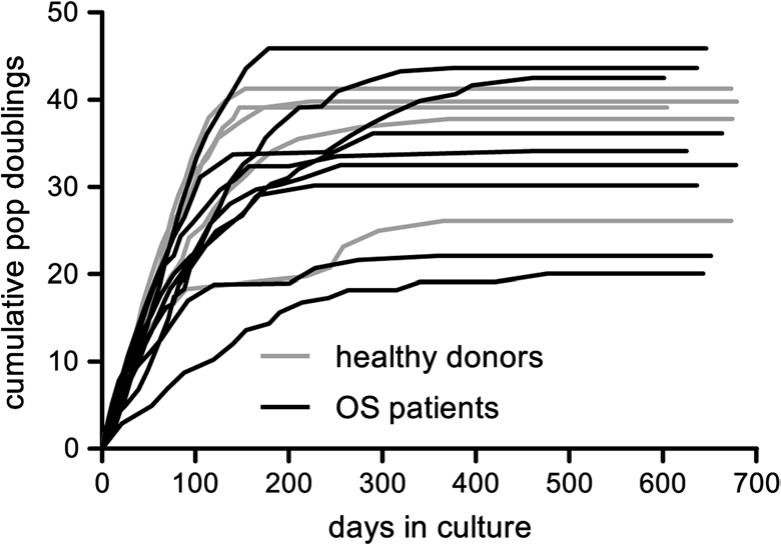
Cumulative population doublings of cultured MSCs from nine osteosarcoma (OS) patients and five healthy donors. All samples were grown in duplicate, averages of each duplicate are shown.

MSCs of nine OS patients and five healthy donors were long term cultured (average number of days in culture 649, range 601–679 days). From each individual sample, duplicate cultures were established. There were no significant differences between growth rate, cumulative population doublings (median cumulative population doublings OS patients 34 vs. healthy donors 39; *P* value Mann–Whitney U test 0.70), passage number at termination of culture (mean passage number OS patients 21 vs. healthy donors 23; *P* value Mann–Whitney U test 0.74) or time to growth arrest (median days to growth arrest OS patients 441 days vs. healthy donors 222 days; *P* value Mann–Whitney U test 0.15) between MSCs of OS patients and healthy donors. The cumulative population doublings are shown in Figure 1 (averages of the duplicate cultures are shown per sample). All cultures exhibited rapid exponential growth in the first few passages. Later, proliferation slowed down and eventually stopped, characteristic of cultured cells in crisis. At termination of the cultures, viable cells were present in all samples. Cultures were terminated at the end of the growth curves. At this point, in none of the cultures there was evidence of cells escaping the crisis by an increase in proliferation and corresponding morphological changes indicative for spontaneous in vitro malignant transformation.

**Figure 2:**
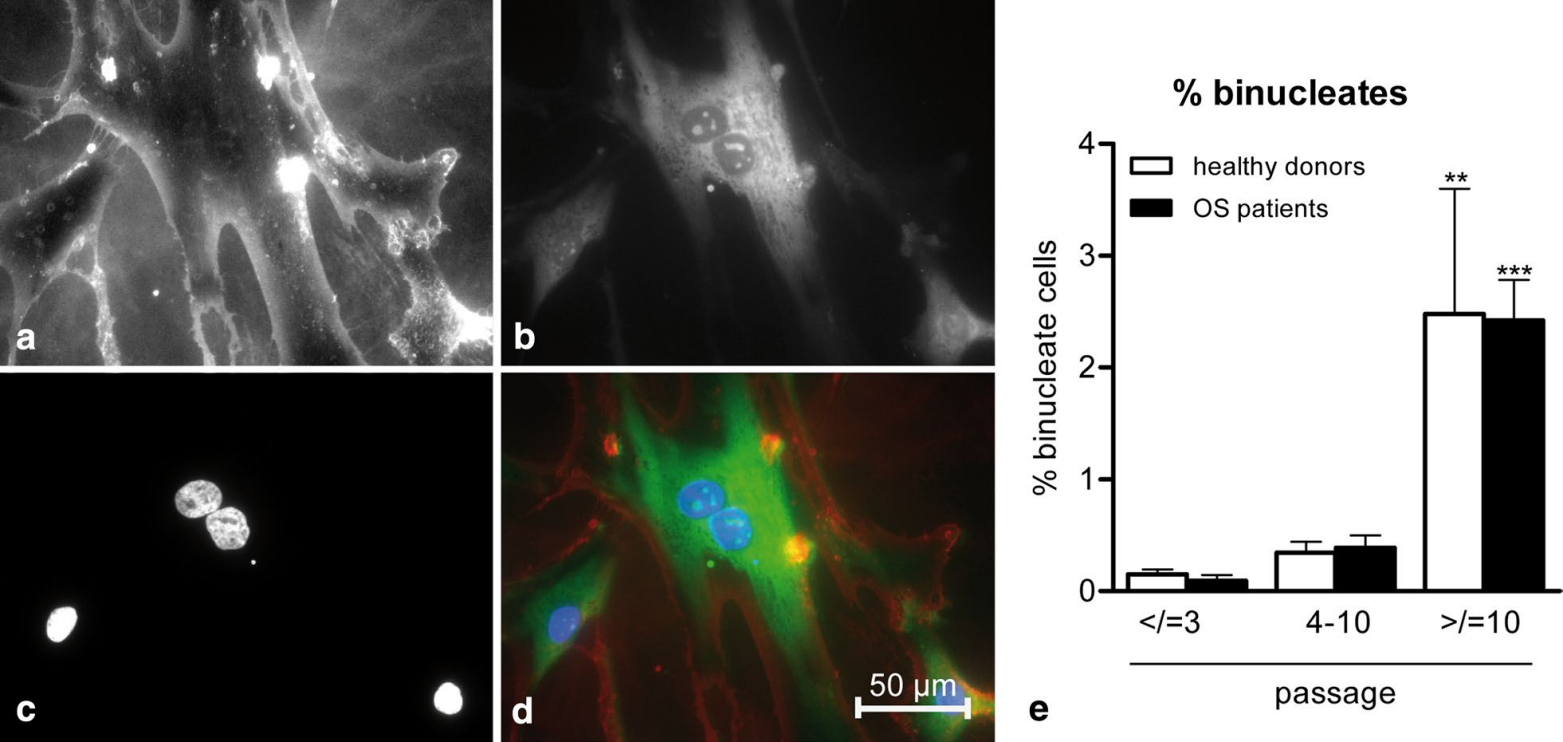
Increased binucleation upon increasing passage number. To facilitate cell staining and counting of nuclei, cells were grown on glass coverslips and stained using wheat-germ agglutinin (**a** cell membranes), acridine orange (**b** cytoplasm) and DAPI (**c** nuclei), shown in ×100 magnification. **d** Overlay with cell membrane in *red*, cytoplasm in *green* and nuclei in *blue*. A representative example of a high passage binucleate cell is shown. Note also the presence of a micronucleus (*panel* **c**). **e** Upon increasing passage number, more binucleate cells were noted, but no differences were seen between healthy donor-derived and osteosarcoma (OS) patient-derived MSCs (Kruskal–Wallis test *P* value <0.0001, Dunn’s post-test compared to early passage cells; P value **<0.01; ***<0.001.

Morphology of the cells was inspected with every passage. Low passage cells were spindle-shaped, higher passage cells were larger. Upon increasing passage number, increasing frequencies of binucleate cells were noted using phase contrast microscopy. To quantify binucleation, cells were grown on coverslips and cytoplasm, cell membranes and nuclei were stained. Mono- and binucleate cells were counted (Figure 2a–d). There was no difference in percentage of binucleate cells between MSCs derived from OS patients and healthy donors (Figure 2e).

### HCLS1 mRNA expression is downregulated in OS patient derived MSCs

**Figure 3:**
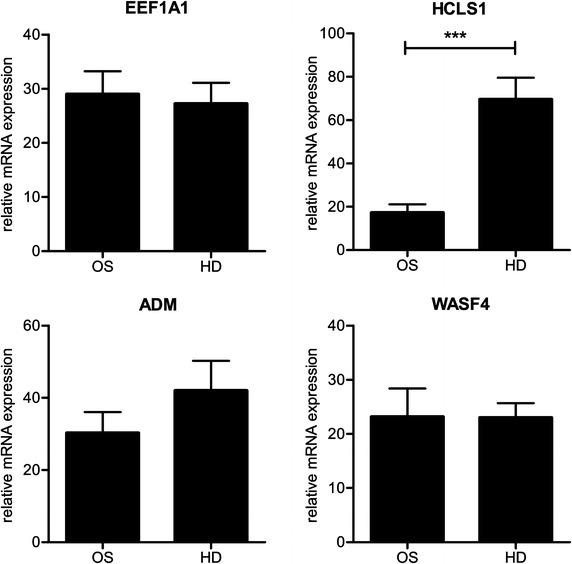
*HCLS1* gene expression is lower in MSCs from osteosarcoma patients as compared to healthy donors. Expression of *HCLS1* as determined by qPCR in bone-marrow derived MSCs no later than passage 5 is lower in MSCs derived from newly diagnosed osteosarcoma patients (OS; n = 14) than in MSCs derived from healthy donors (HD; n = 9). ***P value Mann–Whitney-U test = 0.0005. Expression levels for *EEF1A1*, *ADM* and *WASF4* were not significantly different.

Using microarray gene expression analysis, five genes were found to be differentially expressed between early passage healthy donor derived MSCs (n = 5) and OS patient derived MSCs (n = 7), with a false discovery rate of 20%. Expression of *HCLS1*, *ADM*, *EEF1A1*, *LOC644739* (or *WASF4*) and *LOC441155* was lower in patient derived MSCs as compared to healthy donor derived MSCs. To perform simultaneous technical and biological validation of the microarray results, the original low passage MSC samples were recultured and the original series expanded to now include four additional healthy donor derived MSCs (total of n = 9) and seven additional OS patient derived MSCs (total of n = 14). Four of the differentially expressed genes were validated using qPCR: hematopoietic cell specific Lyn substrate 1 (*HCLS1*), adrenomedullin (*ADM*), eukaryotic translation elongation factor 1 alpha 1 (*EEF1A1*) and *LOC644739* (or WAS protein family, member 4; *WASF4*). *LOC441155* is a pseudogene for which we did not succeed in designing a sufficiently specific and efficient primer pair. In this second, newly cultured and expanded series, expression of *HCLS1* was significantly lower in OS patient derived MSCs as compared to healthy donor derived MSCs (Figure 3, fold change 0.25, *P* value 0.0005). The other genes were not differentially expressed in this series, perhaps because this was a newly expanded batch of cells. We tried to determine if the observed difference in *HCLS1* expression was also true at the protein level. Although HCLS1 protein could be clearly detected in the positive control cell line Jurkat, we were unable to reproducibly detect HCLS1 in MSCs. For five OS patients we had mRNA available from both bone-marrow derived MSCs and the primary tumor. In these samples, expression of *HCLS1* was higher in tumor tissue as compared to MSCs, but there was no correlation between level of expression of *HCLS1* in low passage MSCs and in the corresponding tumor tissue.

## Discussion

In recent years, MSCs have gained increasing interest as a therapeutic modality for immune modulatory and regenerative purposes [1–4]. In order to achieve sufficient numbers of cells for therapeutic utility, MSCs need to be expanded in vitro prior to infusion. Since long-term culture of non-human MSCs can result in oncogenic transformation and the occurrence of sarcomas in the receiving hosts, there is ongoing debate regarding the safety of using cultured MSCs in clinical studies [35]. In the present study, we compared human MSCs derived from OS patients at diagnosis and from healthy donors to answer two questions. First, do human MSCs transform to a malignant phenotype when cultured in vitro, as has been shown for murine MSCs? Second, are MSCs derived from OS patients more likely to transform than healthy donor derived MSCs?

MSCs from patients and controls exhibited similar growth patterns during long-term in vitro culture. During this time, all cultures reached a plateau in proliferation characteristic of cells in crisis. In contrast to what we have previously observed in murine MSCs [9], we never observed escape of this crisis by a rapidly dividing cell population with changed morphology, even after almost 2 years of continuous culture. There were no phenotypical or functional differences between OS patient derived and healthy donor derived MSCs and early passage karyotypes were normal in both groups. Differentiation capacity and expression of specific cell surface markers was similar.

During prolonged culture, progressive shortening of telomeres occurs [36]. This can cause anaphase chromatin bridges, resulting in failed cytokinesis and consequently in binucleate cells [37]. In the context of loss of expression or function of tumor suppressor proteins and corresponding cell cycle checkpoints, tetraploidy and ultimately aneuploidy will occur [28]. Upon increasing passage, increasing frequencies of binucleate cells were noted; both in patient derived MSCs and healthy donor derived MSCs (Figure 2). However, since we did not observe an increase in proliferation in these cells despite the presence of increasingly high frequencies of binucleate cells, cell cycle checkpoints were probably functionally intact, both in MSCs derived from OS patients and from healthy donors.

In addition to the functional read-out of long-term culture, we also performed gene expression analysis on early passage cells. Expression of *HCLS1* was downregulated in MSCs from OS patients as compared to MSCs of healthy donors. *HCLS1* is primarily known for its role in the signaling cascade that follows B cell receptor activation. It is highly expressed in B-cell derived malignancies and is associated with a poor outcome [38, 39]. Further studies are needed to determine if *HCLS1* might have a tumor suppressor function and if its loss of expression in OS patients derived MSCs has any relationship to in vivo tumorigenesis. Although we show no functional difference between healthy donor and OS patient derived MSCs, we do not advocate the use of MSCs from known cancer patients for clinical purposes. The relevance of the differential expression of *HCLS1* remains unknown and there might be other undetected (pre-) malignant alterations.

Both on a transcriptional and on a functional level, OS patient and healthy donor derived MSCs were very similar. There are several possible explanations for the observed similarities between OS patient derived and donor derived MSCs. First, we obtained MSCs from the iliac crest, while all tumors in our series were located at the metaphyseal ends of the long bones. OS occurs at a time and place of active growth and perhaps this pro-proliferative microenvironment (with high expression of growth factors) is an essential prerequisite for oncogenic transformation of MSCs. Second, (pre-) oncogenic alterations may be present in only one or a few local mesenchymal tumor precursor cells and not in MSCs at a distant site such as the iliac crest. This could be due to somatic mosaicism, similar to what has been shown for the enchondromatosis syndromes Ollier disease and Maffucci syndrome [40]. Third, according to the ‘multiple hit hypothesis’, there may not have been sufficient ‘hits’ for in vitro transformation to occur, even if relevant predisposing alterations are present. Ionizing radiation is a well-known risk factor for developing osteosarcoma and low-dose radiation facilitates oncogenic transformation in telomerase-transduced immortalized MSCs [41, 42]. Perhaps one or two additional ‘hits’ (for example loss of cell cycle checkpoint control or radiation-induced DNA damage) would be enough for oncogenic transformation to occur.

Fourth, pre-malignant alterations may have been present in only a few cells, which would not lead to large enough differences in gene expression to be picked up by genome wide expression analysis. Novel techniques to study genetic alterations at a single cell level might be able to pick up these rare events [43, 44]. Finally, although there is compelling evidence to suggest MSCs are the precursor cells for human OS, this has not yet been unequivocally proven.

## Conclusions

During long-term in vitro culture of human OS patient and healthy donor derived MSCs, there was no evidence for neoplastic changes to occur. We could not confirm our hypothesis that MSCs of OS patients might have a higher propensity to oncogenic transformation than healthy donor derived MSCs. In contrast to what has been reported for other species, under the tested conditions, human MSCs do not easily transform. Although we cannot exclude the occurrence of low frequencies of cells with genomic alterations, we did not see a selective growth advantage of aberrant cells nor did we observe karyotypic abnormalities in low passage cells. This data supports the currently held view that administration of low passage cultured healthy donor derived human MSCs for therapeutic purposes is unlikely to result in sarcomas in the host.

## Additional files

Additional file 1:
**Table S1.** Primer sequences used for qPCR experiments. Additional file 2:
**Figure S1.** Differentiation capacity and expression of membranous markers of MSCs. Representative examples are shown (40× magnification). A, chondrogenic differentiation was performed using cell pellet cultures and assessed using toluidine blue staining. Adipogenic differentiation was assessed using phase contrast microscopy (B) and Oil-red-O staining (C). Osteogenic differentiation was assessed using phase contrast microscopy (D) and Alizarin red staining (E). F, all samples expressed CD73, CD90 and CD105 (bold histograms) as determined by flow cytometry (isotype controle staining shown for comparison). Additional file 3:
**Figure S2.** Representative example of karyotypic analysis of early passage MSCs (OS patient derived MSC003OS; 46,XX).

## Abbreviations

ADM: adrenomedullin
EEF1A1: eukaryotic translation elongation factor 1 alpha 1
FBS: fetal bovine serum
FITC: fluorescein isothiocyanate
HCLS1: hematopoietic cell specific lyn substrate 1
MFI: mean fluorescence intensity
MSC: mesenchymal stromal cell
OS: osteosarcoma
PD: population doublings
PE: phycoerythrin
PerCP: peridinin chlorophyll protein
WASF4: WAS protein family member 4
