# Supplementary material for: Mesenchymal stromal cells of osteosarcoma patients do not show evidence of neoplastic changes during long-term culture
Source: Clin Sarcoma Res. 2015 Jun 23;5:16. doi: 10.1186/s13569-015-0031-1 (PMC4477606; doi:10.1186/s13569-015-0031-1)
Supplement: Additional file 1: — Table S1. Primer sequences used for qPCR experiments. [file 13569_2015_31_MOESM1_ESM.docx]

**Additional Table S1** : Primer sequences used for qPCR experiments

| **Gene** | **sequence 5'- 3'** |
| --- | --- |
| *EEF1A1* | F: ACTGGGATGTGCATGTTGAA |
|  | R: TGGACCCTTCCACTCATAGG |
| *ADM* | F: ACTTCGGAGTTTTGCCATTG |
|  | R: AGCGAACCCAGGTACATCAG |
| *HCLS1* | F: CCAGAAGGACCGAGTGGATA |
|  | R: CATGGACTCAAATTTCGCCT |
| *WASF4* | F: CAACCCCAACAGACCCATAG |
|  | R: CCTGACAATGGGAAGAGGAA |
| *CPSF6* | F: AAGATTGCCTTCATGGAATTGAG |
|  | R: TCGTGATCTACTATGGTCCCTCTCT |
| *GPR108* | F: AGATGCCCCTTTTCAAGCTCTAC |
|  | R: GCCATGAGCCAGTGGATCTTG |
| *CAPNS1* | F: ATGGTTTTGGCATTGACACATG |
|  | R: GCTTGCCTGTGGTGTCGC |
