# Supplementary figures and images for: Mesenchymal stromal cells of osteosarcoma patients do not show evidence of neoplastic changes during long-term culture
Source: Clin Sarcoma Res. 2015 Jun 23;5:16. doi: 10.1186/s13569-015-0031-1 (PMC4477606; doi:10.1186/s13569-015-0031-1)

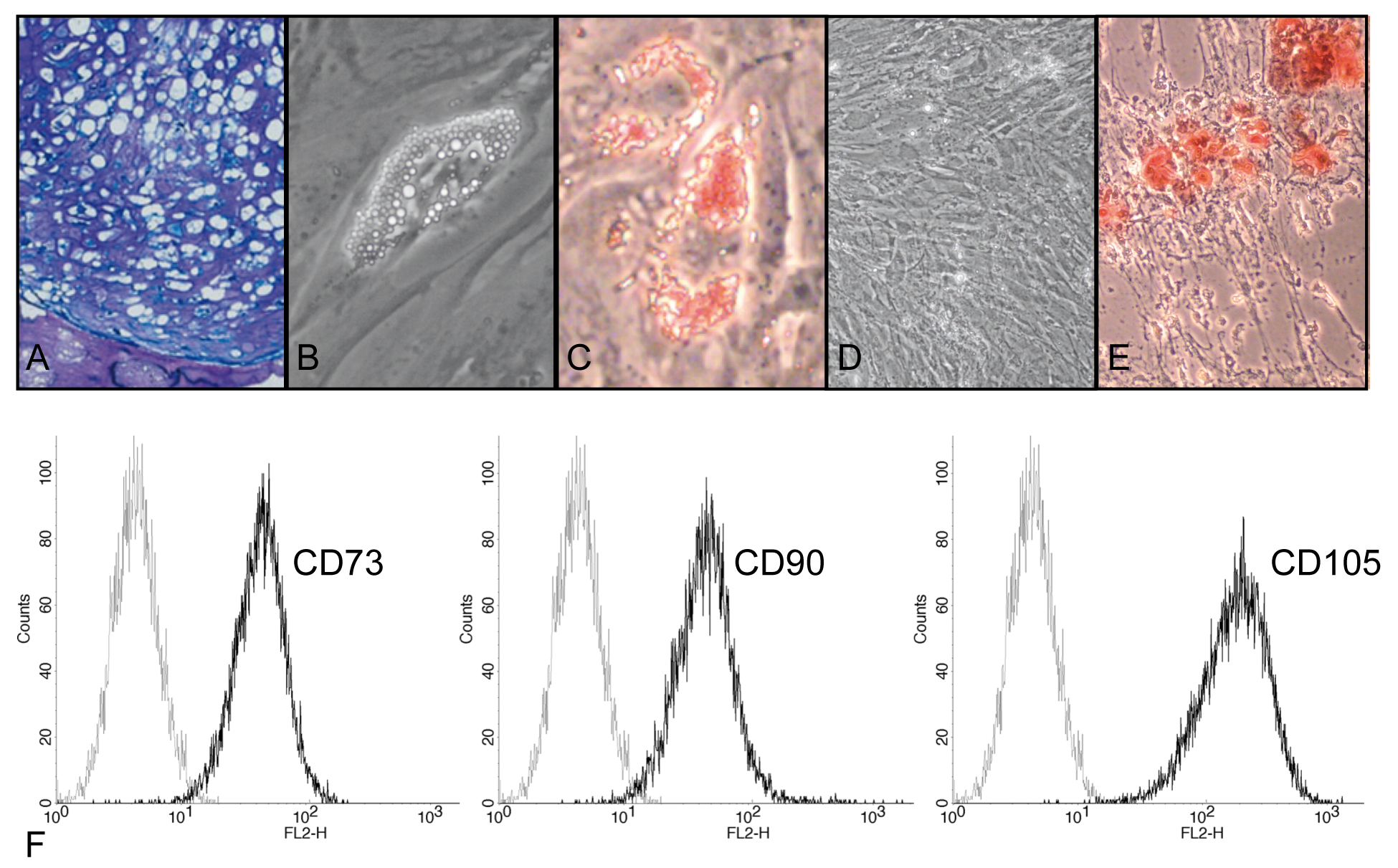

Supplement: Additional file 2: — Figure S1. Differentiation capacity and expression of membranous markers of MSCs. Representative examples are shown (40× magnification). A, chondrogenic differentiation was performed using cell pellet cultures and assessed using toluidine blue staining. Adipogenic differentiation was assessed using phase contrast microscopy (B) and Oil-red-O staining (C). Osteogenic differentiation was assessed using phase contrast microscopy (D) and Alizarin red staining (E). F, all samples expressed CD73, CD90 and CD105 (bold histograms) as determined by flow cytometry (isotype controle staining shown for comparison). [file 13569_2015_31_MOESM2_ESM.tiff]

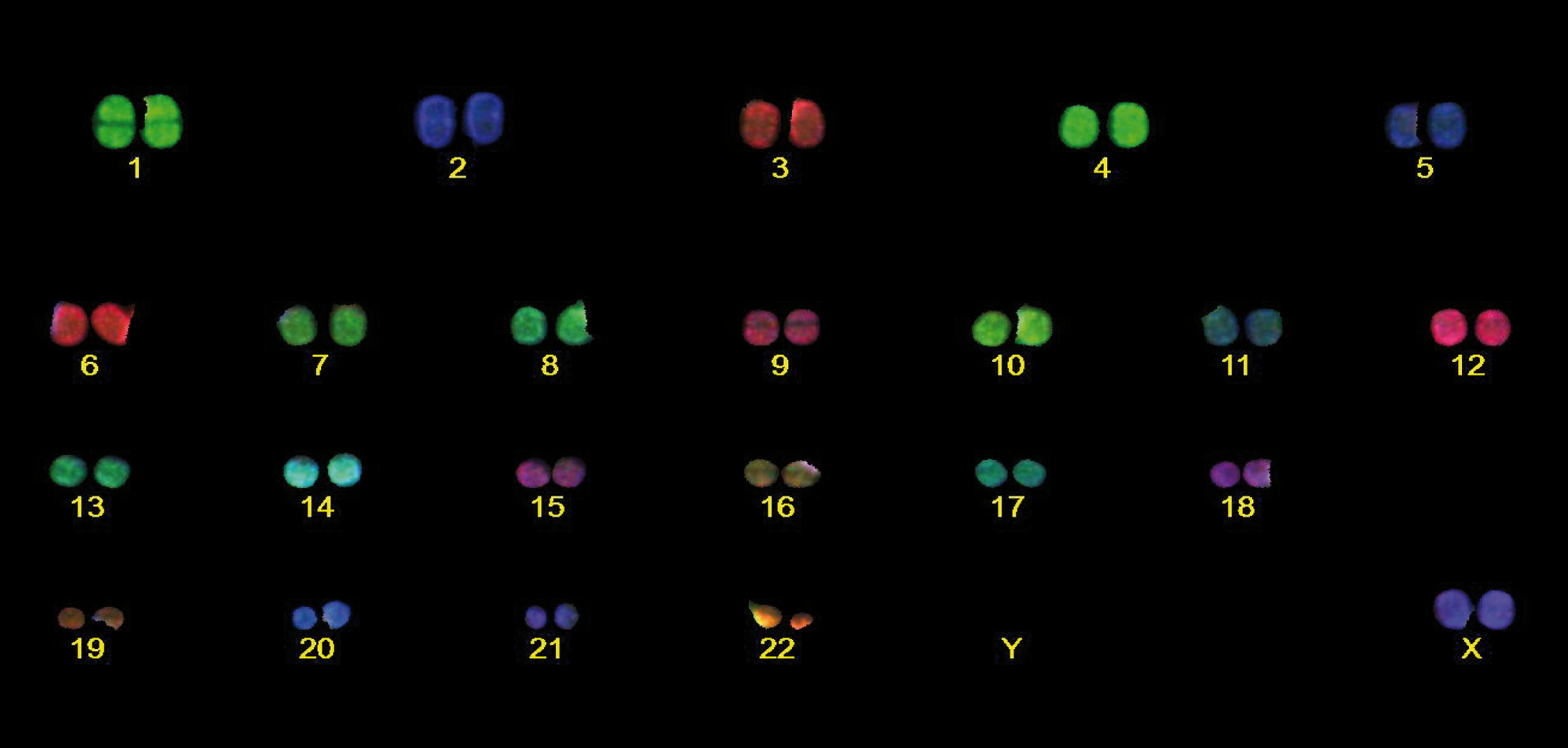

Supplement: Additional file 3: — Figure S2. Representative example of karyotypic analysis of early passage MSCs (OS patient derived MSC003OS; 46,XX). [file 13569_2015_31_MOESM3_ESM.tiff]
